# Salicylic acid-related ribosomal protein CaSLP improves drought and *Pst.DC3000* tolerance in pepper

**DOI:** 10.1186/s43897-023-00054-3

**Published:** 2023-03-14

**Authors:** Huafeng Zhang, Yingping Pei, Qiang He, Wang Zhu, Maira Jahangir, Saeed ul Haq, Abid Khan, Rugang Chen

**Affiliations:** 1 College of Horticulture, Northwest A&F University, Yangling, 712100 Shaanxi China; 2 Department of Horticulture, The University of Agriculture Peshawar, Peshawar, 25130 Pakistan; 3 Department of Horticulture, The University of Haripur, Haripur, 22620 Pakistan; 4 Shaanxi Engineering Research Center for Vegetables, Yangling, 712100 China

**Keywords:** *CaSLP*, Drought tolerance, Salicylic acid, Stomatal, Pepper

## Abstract

The ribosomal protein contains complex structures that belong to polypeptide glycoprotein family, which are involved in plant growth and responses to various stresses. In this study, we found that *capsicum annuum* 40S ribosomal protein SA-like (*CaSLP*) was extensively accumulated in the cell nucleus and cell membrane, and the expression level of *CaSLP* was up-regulated by Salicylic acid (SA) and drought treatment. Significantly fewer peppers plants could withstand drought stress after *CaSLP* gene knockout. The transient expression of *CaSLP* leads to drought tolerance in pepper, and Arabidopsis’s ability to withstand drought stress was greatly improved by overexpressing the *CaSLP* gene. Exogenous application of SA during spraying season enhanced drought tolerance. Ca*SLP*-knockdown pepper plants demonstrated a decreased resistance of *Pseudomonas syringae PV.tomato* (Pst) DC3000 (*Pst.DC3000*), whereas ectopic expression of *CaSLP* increased the *Pst.DC3000* stress resistance in Arabidopsis. Yeast two-hybrid (Y2H) and bimolecular fluorescence complementation (BiFC) results showed that CaNAC035 physically interacts with CaSLP in the cell nucleus. *CaNAC035* was identified as an upstream partner of the *CaPR1* promoter and activated transcription. Collectively the findings demonstrated that *CaSLP* plays an essential role in the regulation of drought and *Pst.DC3000* stress resistance.

**Graphical Abstract:**

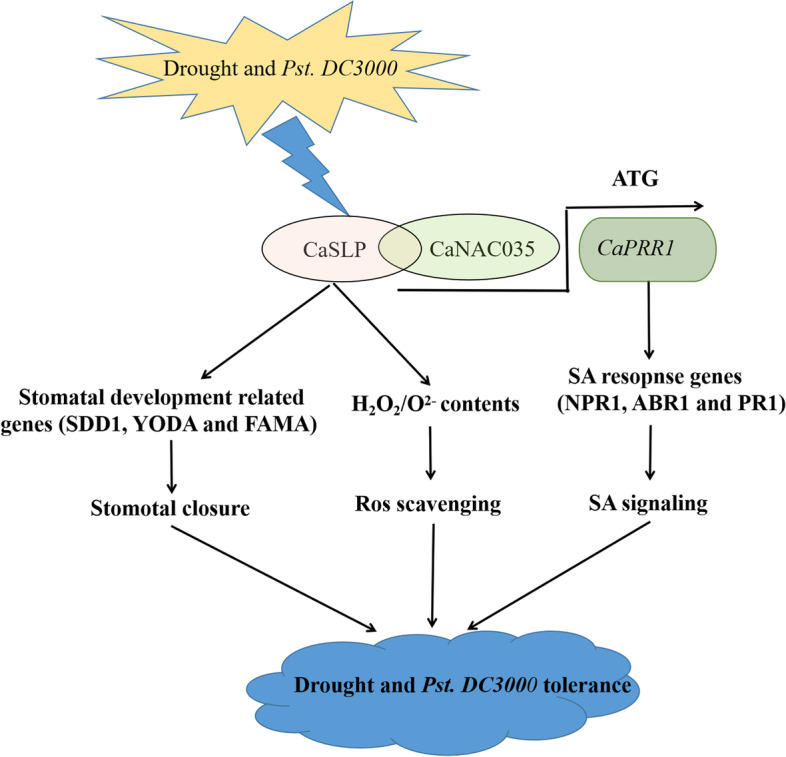

**Supplementary Information:**

The online version contains supplementary material available at 10.1186/s43897-023-00054-3.

## Core

In this study, the transcription factor CaNAC035 interacted with CaSLP in the nucleus, and *CaSLP*-knockdown pepper plants demonstrated a decreased tolerance of drought and *Pst.DC3000* resistance. However, transient expression of *CaSLP* leads to drought and *Pst.DC3000* resistance. And overexpression of *CaSLP* in Arabidopsis increased drought and *Pst.DC3000* resistance. *CaSLP* played a positive role in drought and *Pst.DC3000* stress resistance in pepper.

## Gene & accession numbers

*CaSLP* accession: Capana03g001456, *AtSDD1* accession: At1g04110, *AtYODA* accession: At1g63700, *AtFAMA* accession: At3g24140, *AtTMM* accession: At1g63700, *AtMPK3* accession: At3g45640, *AtNPR1* accession: AT1G64280, *AtPAL3* accession:AT5G04230, *AtICS* accession: AT1G74710, *CaNAC035* accession: Capana05g000569, *CaPR1* accession: LOC107842208.

## Introduction

Drought stress is the most prevalent environmental factor limiting crop productivity (Basu et al. 2016). One of the worst stressors on plants is drought, which causes morphological, physiological, and biochemical alterations, such as decreased growth, gas exchange, photosynthesis, and respiration (Vurukonda et al. 2016). The common consequences of long-term drought as follows. Plant cells accumulate excessive reactive oxygen species (ROS), resulting in damage oxidative and eventually causing cell death. The closure of stomata, which plays a key role in the plant response to drought, decreases water loss of the plant’s leaf. It can also stop the flow of carbon dioxide, which stops photosynthesis (Baxter et al. 2013, Katul et al. 2010). Stomata play an important role in modulating water loss and gas exchange (Dow and Bergmann 2014). The closing process is influenced by the expension degree of stomatal guard cells. The influx of ions and sucrose promotes the absorption of water, causing the swelling of guard cells and forcing the opening of stomatal. Their efflux reduces the osmotic potential of the cell, causing the cell and closure of the stomata (Kim et al. 2010; Lu et al. 1995; Pandey et al. 2007). The transcription factor *ZmNAC49* enhances drought tolerance by reducing stomatal density in maize (Xiang et al. 2021). *AtWRKY1* modulates stomatal movement under drought stress (Qiao et al. 2016), MADS-box factor *AGL16* negatively modulates drought stress via stomatal density in Arabidopsis (Zhao et al. 2020).

Ribosomal proteins have different complex structures in prokaryotes and eukaryotes. Eukaryotic ribosomes are composed of two unequal subunits (60S and 40S), four ribosomal Rnas (RRnas), and 82 distinct RPs. 40S ribosomal protein SA (Laminin-1 receptor) is a 67 kDa monopeptide glycoprotein with a high affinity for Laminin-1 glycoprotein in the basement membrane. It is located or distributed on the surface of different types of cells. The Laminin-1 receptor (40S ribosome protein SA) is a 40S extracellular matrix (ECM) protein involved in cell adhesion and dispersion. Many ribosomal proteins in eukaryotes are involved in cellular processes, in addition to the synthesis of ribosome structures, several ribosomal proteins in eukaryotes are engaged in cellular functions (Warner et al. 2009). Ribosomes are highly conserved proteins that are essential for cell activity. Although its primary or major function is protein synthesis, new in-depth studies have revealed that it is also play a role in cell growth, division and development, as well as in gene regulation (Barakat et al. 2001; Rogalski et al. 2008; Petibon et al. 2016). It was found that the ribosome is an organelle composed of one large and one small subunit in both prokaryotic and eukaryotic cells (Wang et al. 2013). Ribosomal protein can enhance stress tolerance. For example, the expression of the ribosomal protein large subunit gene, *RPL23A*, led to an obvious increase in fresh weight and proline contents under drought and salt stress conditions (Garbarino and Belknap 1994). SA-related cotton (*Gossypium arboreum*) ribosomal protein GaRPL18 contributes to resistance to *Verticillium dahliae* (Gong et al. 2017). Ribosomal protein AgRPS3aE plays a vital role in improving salt tolerance in crops (Liang et al. 2015).

Salicylic acid (SA) is a naturally existing multifunctional phytohormone that can function as a growth regulator. It is involved in a variety of physiological processes, has diverse effects on tolerance to abiotic stress factors, and plays a key role in defence mechanism and drought responses (Hayat et al. 2010). Reports have highlighted the role of SA in inducing stress tolerance in plants. Exogenous salicylic acid boosts tolerance to diverse abiotic stressors, primarily by enhancing antioxidative ability. It also enhances resistance under water deficit conditions and eliminates drought stress (Horváthet al. 2007; Odjegba and Adeniyi 2012). Plant response to exogenous SA depends on the variety, developmental stage, application concentration, application mode, and endogenous SA level (Miura and Tada 2014). Foliar applications of various plant hormones play an important role in drought tolerance under different plant growth stages (Sohag et al. 2020). For instance, 2 mM exogenous SA improves *Impatiens walleriana* drought tolerance (Antonić et al. 2020). Foliar application of SA can enhance the drought tolerance of wheat by reducing ROS accumulation (Shemi et al. 2021). SA reportedly enhances the drought tolerance of wheat by improving proline contents and enzyme activities (Sharma et al. 2017).

NAC (NAM, ATAF1/2, and CUC2) proteins are the plant-specific transcription factors (TFs), and play an important role in plant response to abiotic stresses. In our previous study, we cloned the pepper NAC transcription factor *CaNAC035* gene which belongs to the ATAF subfamily, and found that *CaNAC035* positively regulates cold, salt, and drought stress tolerance (Zhang et al. 2020). Using yeast two hybridization, we screened that 18 proteins that may interact with CaNAC035, and predicted that 18 proteins are involved in several different biological processes (Zhang et al. 2020). Furthermore, we found that the *capsicum annuum* 40S ribosomal protein SA-like (CaSLP) is a CaNAC035-interacting protein in pepper. Pepper *CaSLP* positively regulated drought and *Pst.DC3000* tolerance. *CaSLP* encodes 40S ribosomal protein subunits, which are important for intracellular protein biosynthesis. Previous research has revealed that certain ribosomal proteins can suppress tumors and congenital disorders in humans, as well as play a role in plant resistance and defense.

## Results

### Sub-cellular localization and expression of CaSLP

**Fig. 1:**
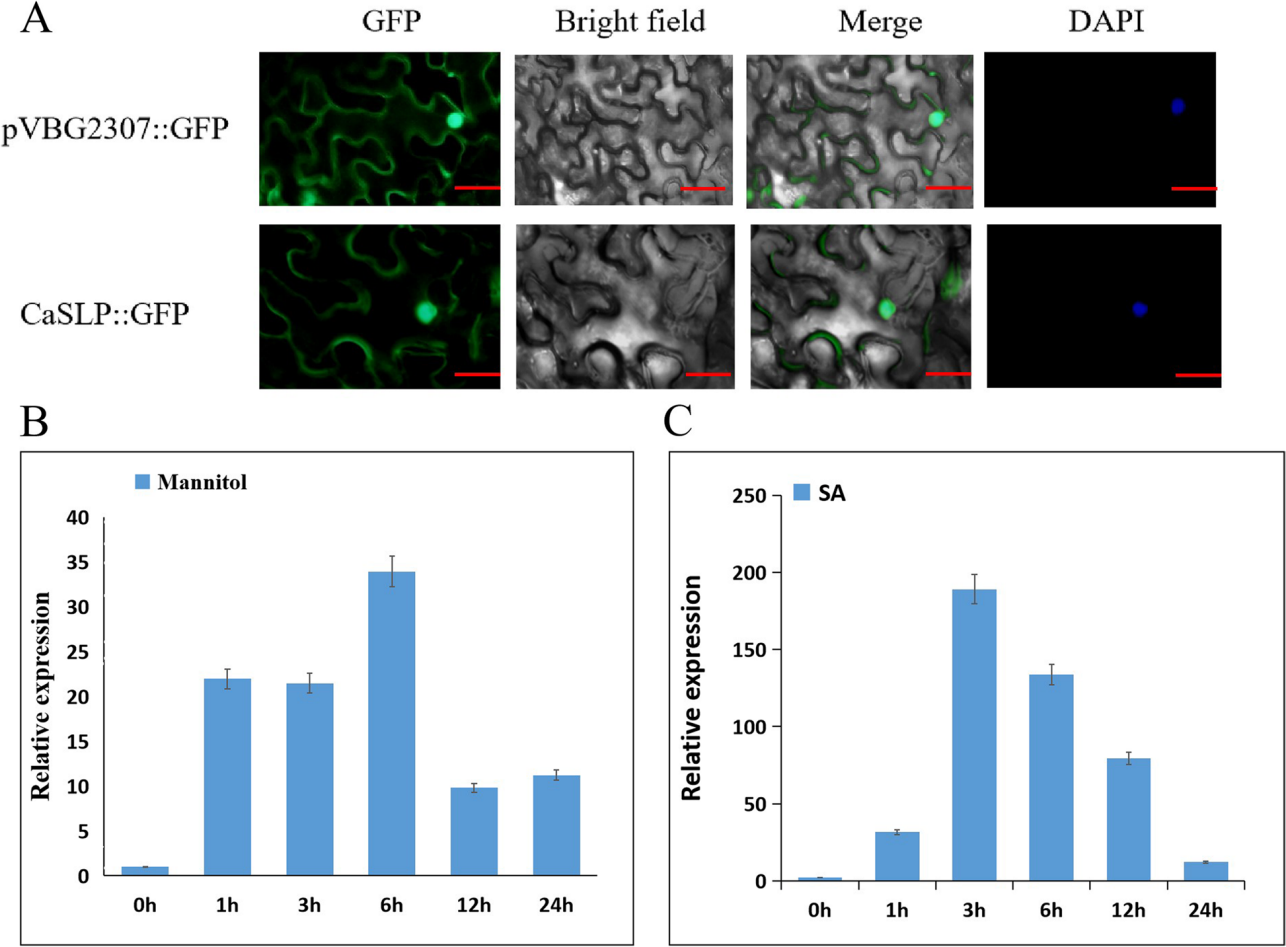
Subcellular localization of CaSLP and the expression pattern of *CaSLP* were induced by salicylic acid and drought. **A** Green fluorescent protein (GFP) control vector (35S:GFP) or CaSLP-GFP fusion protein (35S:CaSLP-YFP) was transiently expressed in *Nicotiana benthamiana* leaves. Fully automatic microscopic fluorescence images were acquired under green fluorescence and bright field. Scale bars, 50 μm. **B** *CaSLP* transcript levels after drought treatment. The pepper leaves were taken at the 0, 1, 3, 6, 12, and 24 h time points for transcript level analysis. **C** *CaSLP* transcript levels under SA treatment. Leaves were acquired at 0, 1, 3, 6, 12, and 24 h time points. Actin was chosen as a control. Error bars show ± SD (*n* = 3)

To experimentally analyze the subcellular localization, the full length of CaSLP was inserted into the green fluorescent protein (GFP) reporter gene, and the fusion construct 35S:CaSLP:GFP and 35S::GFP were transiently expressed in tobacco leaves. The nuclei were stained with DAPI. *N. benthamiana* leaves were collected efficiently. Fluorescent signaling of the 35S:CaSLP:GFP fusion protein was was distributed throughout the cell nucleus and cell membrane of tobacco epidermal cells, showing that CaSLP was localized in the cell nucleus and cell membrane (Fig. 1A). The qRT-PCR was performed to characterize the expression pattern of *CaSLP.* After moisture stress, the expression of *CaSLP* was upregulated within 6 h (Fig. 1B). At 3 h after exogenous SA treatment, the expression of *CaSLP* progressively peaked (Fig. 1C). These results indicated that *CaSLP* is primarily induced by drought and SA.

### Enhanced drought tolerance in pepper plants silenced for *CaSLP*

**Fig. 2:**
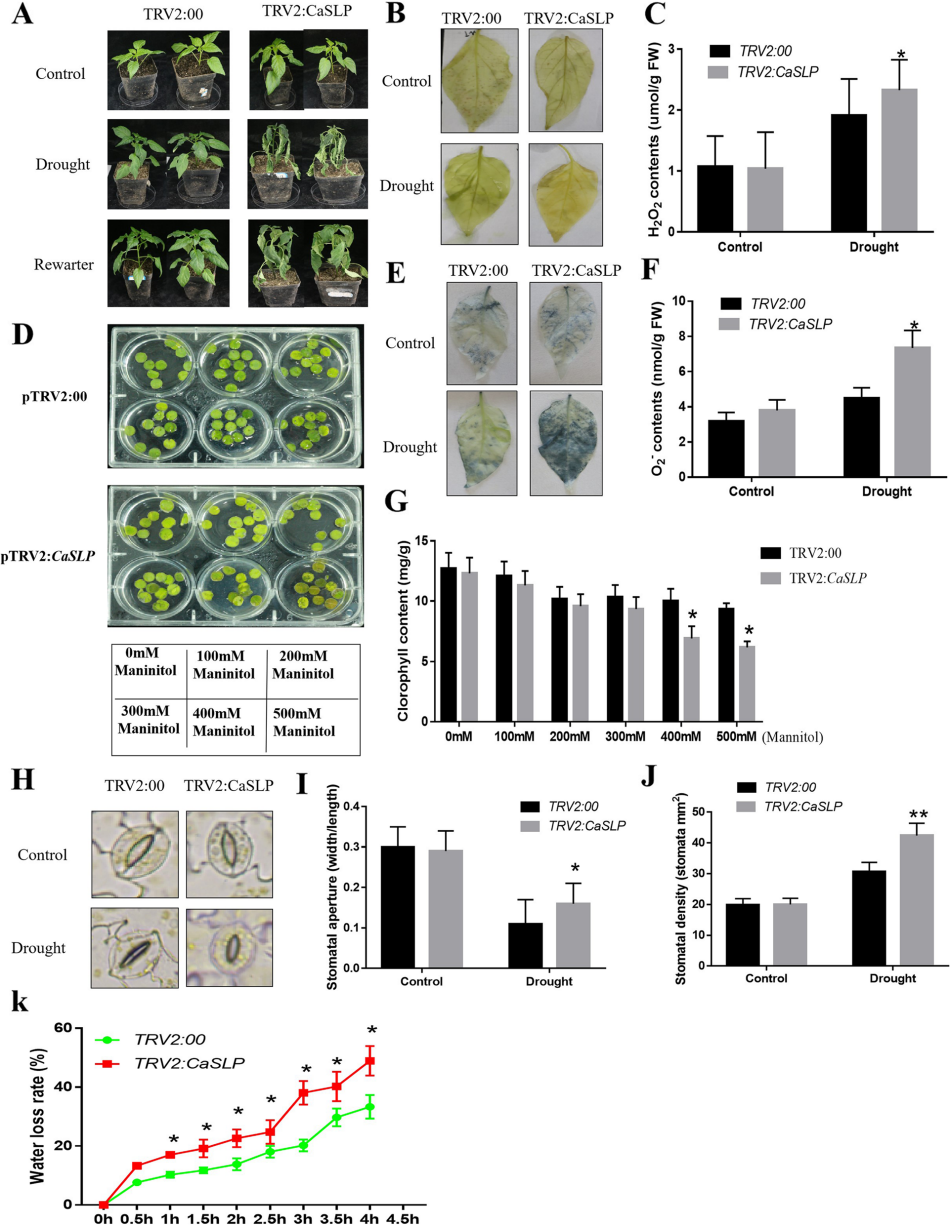
Silencing of *CaSLP* by virus-induced gene silencing (VIGS) decreases drought tolerance in pepper. **A** Phenotype of *CaSLP*-silenced and control (TRV, tobacco rattle virus) plants before and after 15 d of drought treatment. Plants were treated under water deficit conditions for 15 days, then rewatered for 3 days. **B**, **E** Histochemical staining of 3,3’-diaminobenzidine (DAB) and nitro blue tetrazolium (NBT) was performed to detect the accumulation of H_2_O_2_ and O_2_^.−^ levels, respectively. **C**, **F** H_2_O_2_ and O_2_.^.−^ contents. **G** Chlorophyll contents of the *CaSLP*-silenced and control plants before and after 15 d of drought treatment. **H** Photomicrographs of stomata from the *CaSLP*-silenced and control plants. **I** The stomatal aperture was analyzed under the microscope. **J** Stomatal density **K** Water loss rate of the *CaSLP*-silenced and control plants. Error bars represent ± SD (*n* = 3). Asterisks represent a significant difference between *CaSLP*-silenced and control plants under the same condition (*, *P* < 0.05; **, *P* < 0.01)

*CaSLP* upregulation in response to drought revealed that it plays a function in drought stress response. VIGS studies were carried out to investigate this possibility.. As indicated in supplemental Figure S1, TRV2-treated plants: *CaPDS* solution revealed photo-bleaching in the leaves, confirming that the treatment was successful. Then, the silencing efficiency was measured through qRT-PCR, which was almost 85%. (Figure S1). No obvious morphological differences were found under normal growth conditions. Under drought stress, the *CaSLP*-silenced pepper plants showed a drought-resistant phenotype compared with control pepper plants (Fig. 2A). The results of the measurement of H_2_O_2_ and O_2_^.−^ contents, the ROS indicators showed that the H_2_O_2_ and O_2_^.−^contents in the *CaSLP*-silenced pepper plants were significantly higher than those in the control pepper plants after drought treatment (Fig. 2C, F). The DAB and NBT staining of *CaSLP*-silenced pepper exhibited stains much darker and deeper than in the control pepper plants when plants were exposed to drought stress (Fig. 2B, E). When the plants were exposed to different mannitol concentrations, the *CaSLP*-silenced pepper showed more severe leaf wilting than did the control under each mannitol concentration (Fig. 2D). *CaSLP*-silenced pepper plants had lower chlorophyll content than the control (Fig. 2G). In addition, the stomatal aperture and water loss rate were measured. No apparent differences were found between the *CaSLP*-silenced and control cells under normal conditions. However, when subjected to drought, the VIGS plants showed slightly higher stomatal aperture, stomatal density and water loss rates than the control plants (Fig. 2H-K). All of these data demonstrated that silencing of *CaSLP* reduces drought tolerance.

### Transient expression of *CaSLP* improved pepper drought tolerance

**Fig. 3:**
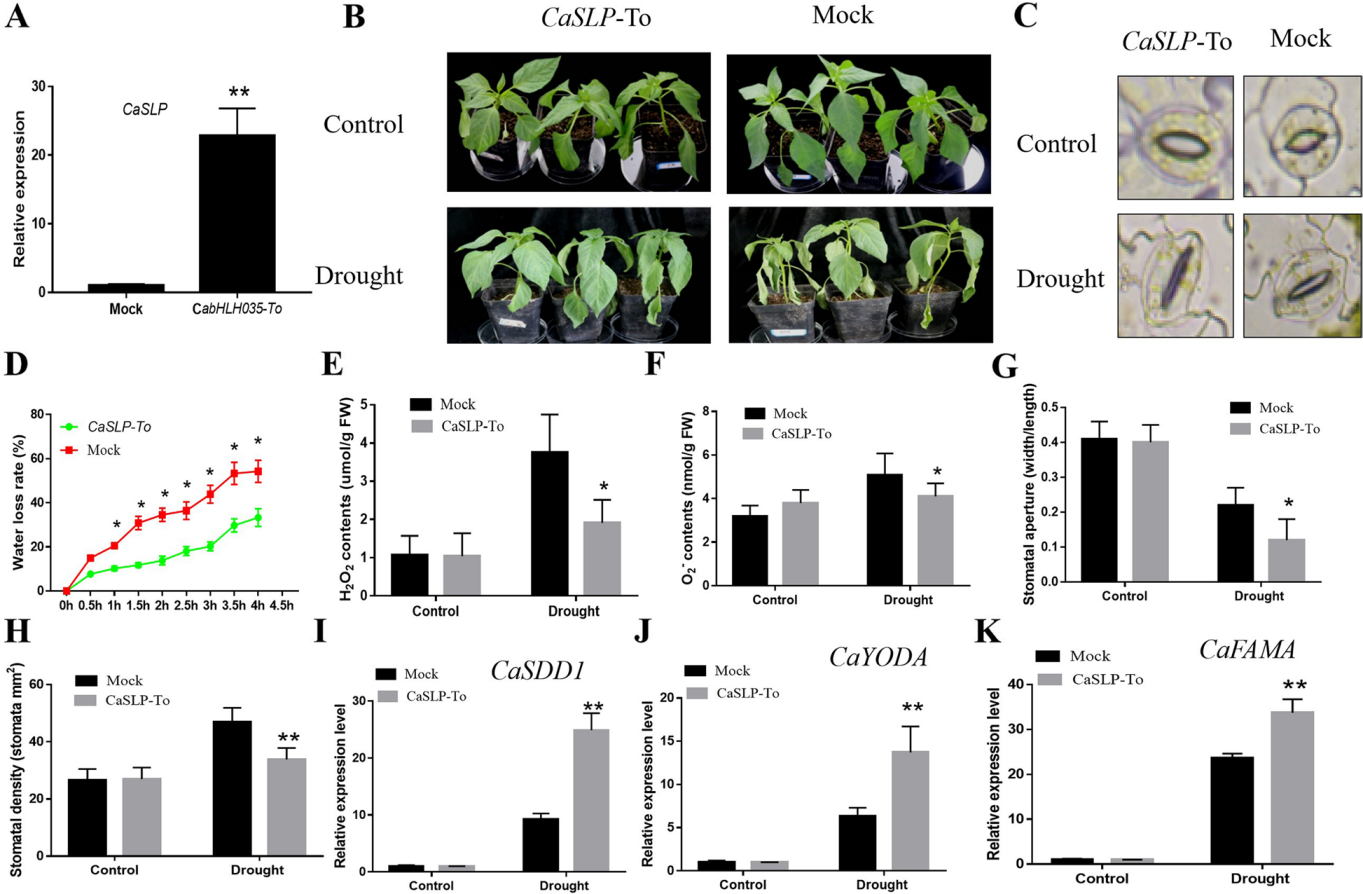
Transient expression of *CaSLP* significantly improved pepper drought tolerance. **A** The transcript levels of *CaSLP.* **B** Phenotype of *CaSLP*-To (Transient overexpression) and the control (35S::GFP) plants before and after 48 h of drought treatment. Plants were treated under water deficit conditions for 48 h. **C**-**G** Stomatal aperture (**C**), Water loss rate (**D**), H_2_O_2_ content (**E**), O_2_.^.−^ content (**F**), Contents of *CaSLP*-To and the control plants before and after 48 h of drought treatment. **G** Stomatal aperture. **H** Stomatal density. **I**-**K** Transcript levels of the stomatal development genes *SDD1* (**H**), *YODA* (**I**), and *FAMA*(**J**) were measured by qRT-PCR in *CaSLP*-To and the control plants before and after the drought treatment. Values are means ± SD (*n* = 3). Different asterisks represent significant differences (*, *P* < 0.05; **, *P* < 0.01)

To explore the function of *CaSLP* in response to drought stress tolerance, transient overexpression of *CaSLP* (*CaSLP*-To) in pepper was performed. The *CaSLP*-To and Mock plants were exposed to drought for 48 h. *CaSL*P-To expression was assessed. It was clearly taller than Mock pepper plants (Fig. 3A). The *CaSLP*-To plants maintained good growth and leaf turgor. Without stressful conditions, no clear differences were found in plant morphology between *CaSLP*-To and Mock pepper plants. However, drought stress caused severe leaf wilting and a significant increase in ROS levels. The H_2_O_2_ and O_2_^.−^ contents of *CaSLP*-To plants were significantly lower than that of Mock pepper plants (Fig. 3B, E, F). Next, we measured the stomatal aperture. The stomatal aperture of *CaSLP*-To and Mock plants did not differ (Fig. 3C). Upon the drought exposure, the stomatal aperture of *CaSLP*-To plants showed a lower stomatal aperture and stomatal density compared with the control Mock pepper plants (Fig. 3G, H). Water loss rate was also assessed and observed that *CaSLP*-To plants lose less water than Mock pepper plants (Fig. 3D). We also detected the transcript levels of the stomatal development-related genes *SDD1, YODA*, and *FAMA* in *CaSLP*-To and the control plants. The transcript abundance of *SDD1, YODA*, and *FAMA* in *CaSLP*-To plants were higher than control plants (Fig. 3I-K). *CaSLP* transient expression increased drought stress tolerance. These findings showed that *CaSLP* transient expression improved drought stress tolerance.

### Overexpression of *CaSLP* in Arabidopsis increased drought tolerance

**Fig. 4:**
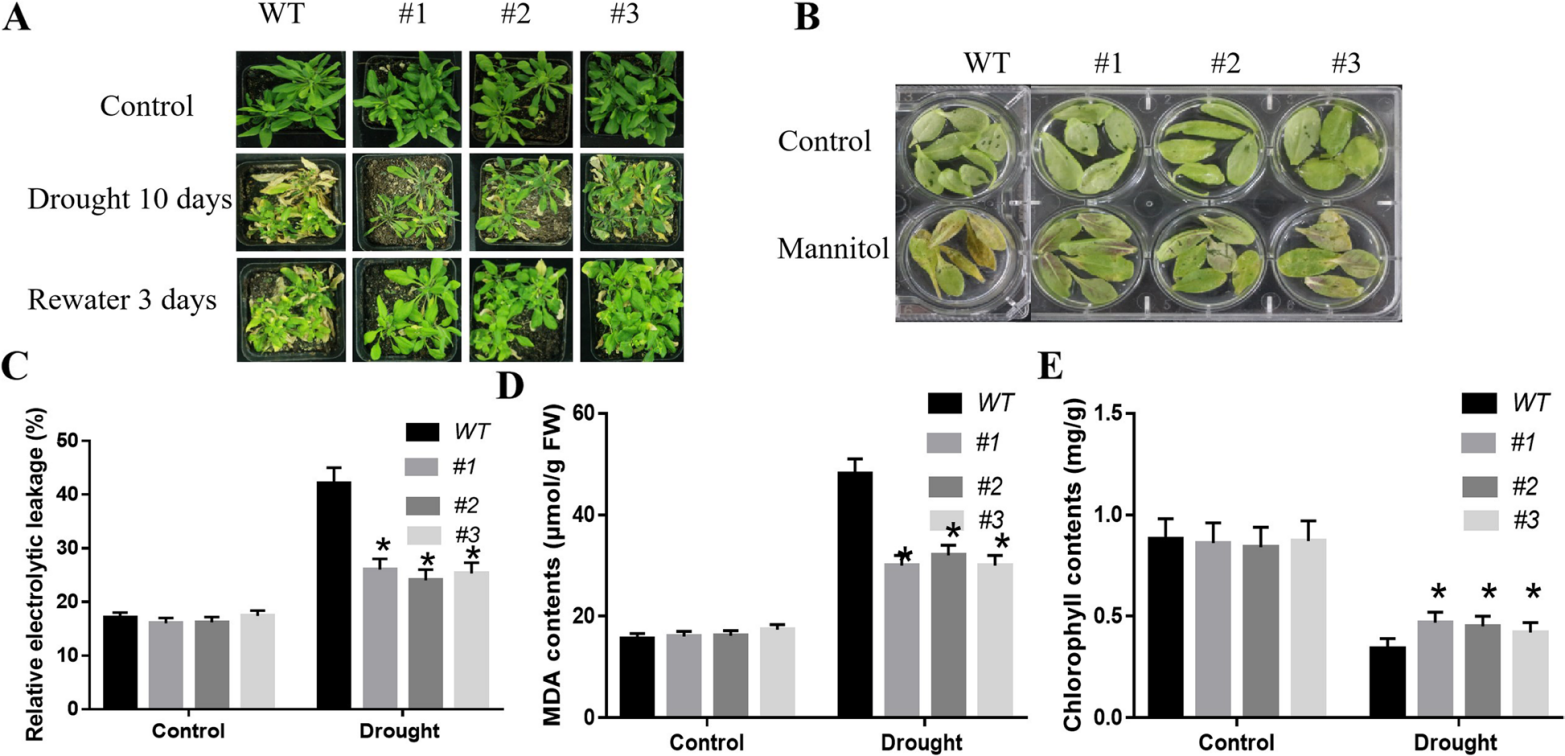
Overexpression of *CaSLP* confers enhanced drought tolerance to transgenic Arabidopsis. **A**-**B** The phenotype of transgenic and wild-type (WT) plants before and after 10 d of drought treatment. Plants were treated under water deficit conditions for 10 d, then rewatered for 3 d. **C**-**E** Relative electrolyte leakage (**C**), and Malondialdehyde (MDA) content (**D**), Chlorophyll content (**E**) of the transgenic and WT plants before and after the drought treatment. Values are means ± SD (*n* = 3). Asterisks show a significant difference between the transgenic lines and WT under drought stress (*, *P* < 0.05; **, *P* < 0.01)

To further determine the function of *CaSLP* in drought tolerance, *Agrobacterium*-mediated transformation was performed to conduct 35 s:*CaSLP*:GFP transgenic overexpression (OE) lines in Arabidopsis. A obvious upregulated in *CaSLP* expression level was confirmed in the OE lines than WT plants. *CaSLP* transgenic lines #1, #2, and #3 were chosen to study the roles of *CaSLP* in drought tolerance. Under well-watered conditions, no apparent difference was found between the *CaSLP* transgenic and the wild type (WT). After drought stress, the WT showed more severe leaf wilting than the *CaSLP* transgenic lines (Fig. 4A, B). Relative electrolyte leakage (REL), malondialdehyde (MDA), and chlorophyll contents supported better growth performance of *CaSLP* transgenic lines following drought exposure. The *CaSLP* transgenic lines demonstrated lower REL and MDA contents, and greater chlorophyll content than WT (Fig. 4C-E). These findings showed that *CaSLP* overexpression in Arabidopsis significantly increased drought tolerance.

### Exogenous spraying salicylic acid improved *CaSLP*-silenced pepper plant drought tolerance

**Fig. 5:**
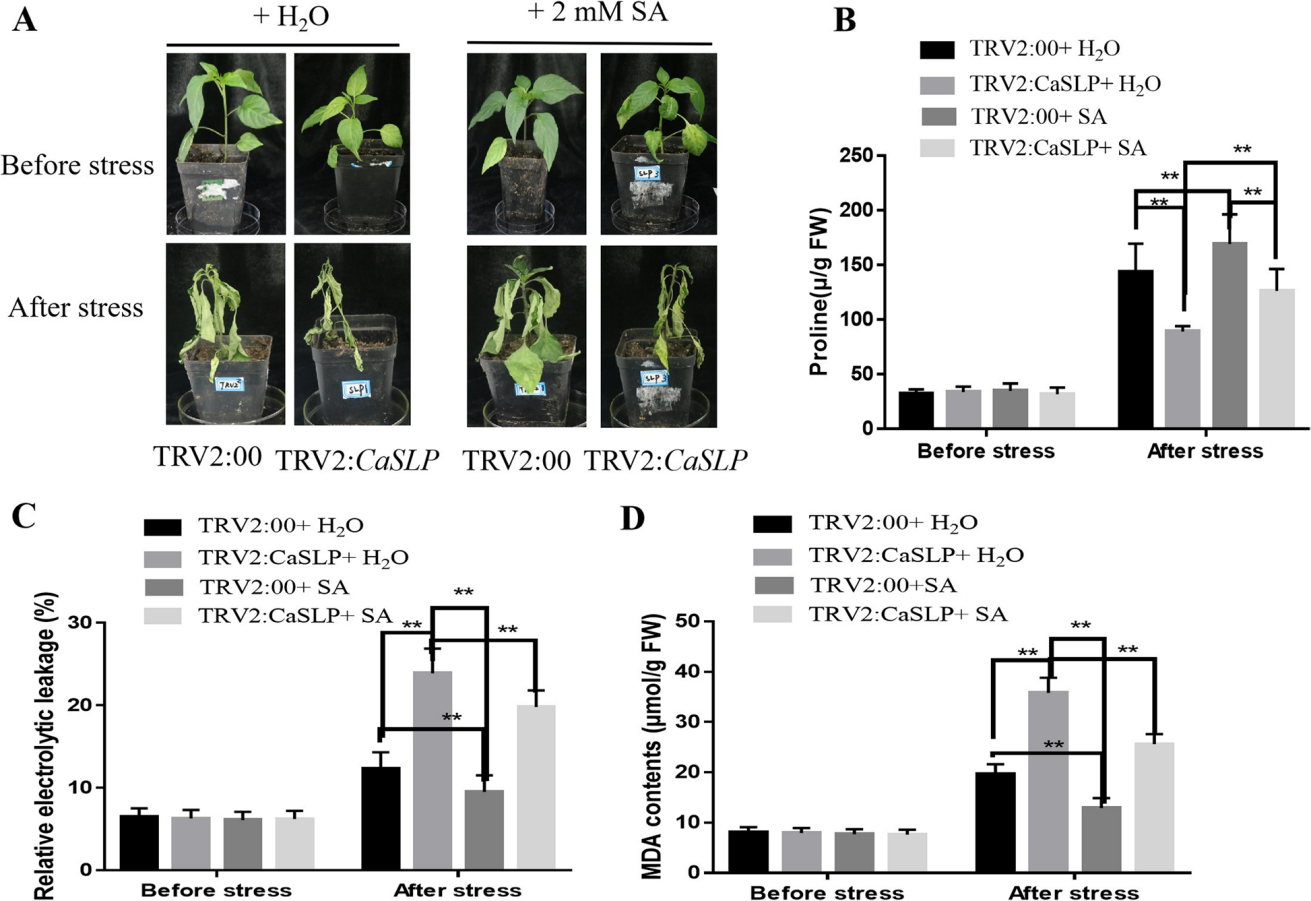
Exogenous spraying salicylic acid enhanced the drought tolerance of *CaSLP*-silenced plants. **A** Phenotypes of the VIGS line (TRV-*CaSLP*) and TRV control before and after exogenous spraying salicylic acid treatment. **B**-**D** Proline content (**B**), relative electrolyte leakage (**C**), and malondialdehyde (MDA) content (**D**) of the VIGS line (TRV-*CaSLP*) and TRV control plants. Values are means ± SD (*n* = 3 replicates). Asterisks represent a significant difference between the transgenic lines and WT under drought stress (*, *P* < 0.05; **, *P* < 0.01)

Drought has become a dangerous threat to decrease crop yields. Exogenous spraying SA regulator has been effectively used to reduce drought tolerance in field crops. We anticipate *CaSLP*-mediated drought tolerance via a salicylic acid route based on the visible induction of *CaSLP* in response to drought (Fig. 1). Therefore, the *CaSLP*-silenced pepper and control plants were sprayed exogenously with 2 mM SA (Fig. 5A). No noticeable differences were found in proline, REL, and MDA contents between the *CaSLP*-silenced and control plants in the absence of stressful circumstances. However, after exogenous spraying SA treatment, the *CaSLP*-silenced pepper plants exhibited lower REL, MDA, and lower proline contents in comparison with the plants that were treated with water (Fig. 5B-D). These above-mentioned data demonstrate that exogenous application of salicylic acid drastically enhanced drought tolerance and further showed the importance of salicylic acid in *CaSLP*-regulated drought tolerance.

### Altered expression of stomatal development-related genes

**Fig. 6:**
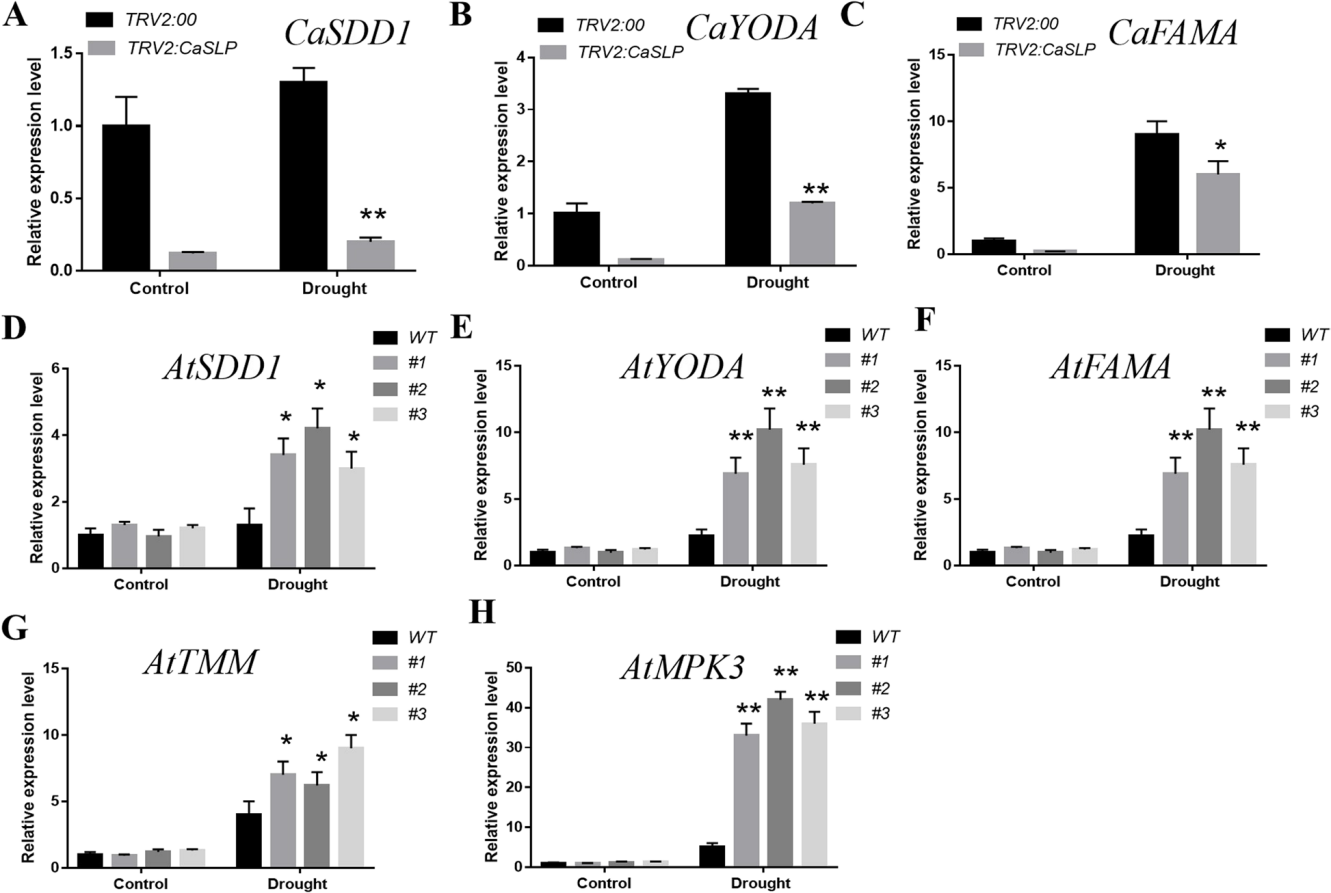
*CaSLP* affects the expression levels of stomatal development genes. **A**-**C** The transcript levels of stomatal development-related genes, including *SDD1*, *YODA,* and *FAMA* were analyzed by qRT-PCR in VIGS line (TRV-*CaSLP*) and TRV control plants, **D**-**H** *AtSDD1*, *AtYODA*, *AtFAMA*, *AtTMM*, and *AtMPK3* were analyzed by qRT-PCR in transgenic lines and wild-type (WT) plants. Values are means ± SD (*n* = 3 replicates). Asterisks indicate a significant difference between the transgenic lines and WT under drought stress (*, *P* < 0.05; **, *P* < 0.01)

To further explore the underlying mechanism of enhanced tolerance to drought stress of *CaSLP*, the expression profiles of several known stomatal development-related genes were selected after drought treatment. We measured stomatal development-related genes in pepper, including *SDD1*, *YODA,* and *FAMA*. The qRT-PCR results showed that the expressions of *SDD1*, *YODA,* and *FAMA* were decreased in *CaSLP*-silenced cells than in control cells (Fig. 6A-C), suggesting that *CaSLP* acts as a positive regulator of drought signaling. We also measured the expression levels of stomatal development-related genes in Arabidopsis (*AtSDD1*, *AtYODA*, *AtFAMA*, *AtTMM,* and *AtMPK3*) after exposure to drought stress. The expressions of *AtSDD1*, *AtYODA*, *AtFAMA*, *AtTMM*, and *AtMPK3* were greater in transgenic plants than in WT plants (Fig. 6). These results indicate that overexpression of *CaSLP* increases the drought tolerance of Arabidopsis.

### *CaSLP*-knockdown pepper plants demonstrated decreased *Pst.DC3000* resistance

**Fig. 7:**
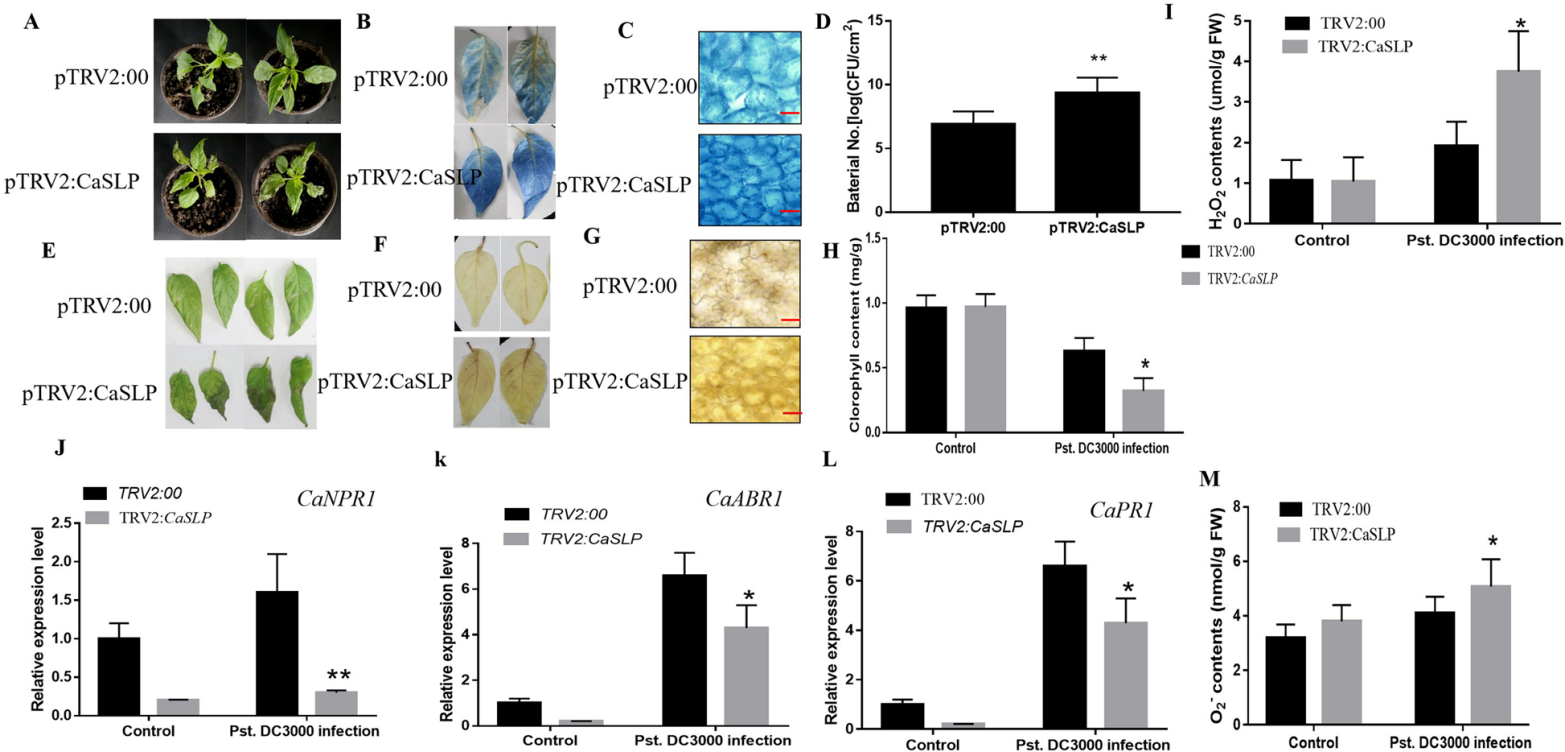
Silencing of *CaSLP* by virus-induced gene silencing (VIGS) decreases *Pst.DC3000* resistance in pepper. **A**, **E** Disease symptoms of *CaSLP*-silenced and control (TRV, tobacco rattle virus) plants before and after the *Pst.DC3000* infection. Plants were infected with *Pst.DC3000* for 3 days. **B**, **F** Histochemical staining with 3,3’-diaminobenzidine (DAB) and Trypan blue for analyzing the accumulation of H_2_O_2_ in the *CaSLP*-silenced and control plants before and after 3 d of *Pst.DC3000* infection. **C**, **G** Trypan blue and DAB staining for cell death in the *CaSLP*-silenced and control plants before and after *Pst.DC3000* infection. Bar = 50 μm. **D**-**I** Bacterial numbers (**D**), Chlorophyll content (**H**), and H_2_O_2_ content (**I**) of the *CaSLP*-silenced and control plants before and after 3 d of *Pst.DC3000* infection. **J**-**L** The expression of the SA response genes of *CaNPR1, CaABR1* and *CaPR1*. M, O_2_^.−^ content of the *CaSLP*-silenced and control plants before and after *Pst.DC3000* infection. Error bars represent ± SD (*n* = 3). Asterisks indicate a significant difference between *CaSLP*-silenced and control plants(*, *P* < 0.05; **, *P* < 0.01)

To examine the *Pst.DC3000* susceptibility, the *CaSLP*-silenced plants and control leaves were infected with *Pst.DC3000*. 10 mM MgCl_2_ (control treatment) and *Pst.DC3000* suspensions were injected into the plant leaves. After *Pst.DC3000* treatment, the leaves of *CaSLP*-knockdown plants was more yellow and wilted than the control. This finding was indicated by the lower chlorophyll contents of *CaSLP*-knockdown plants (Fig. 7A, E, H). Additionally, to explore the accumulation of reactive oxygen species (ROS) post-*Pst.DC3000* stress in the control and silenced pepper plants, the trypan blue and DAB staining of the leaf samples were performed. Figure 7B, F showed that as compared with the control, the accumulation of H_2_O_2_ and O_2_^.−^ contents of *CaSLP*-silenced plants was markedly higher (Fig. 7I, M). These results showed that the *CaSLP*-silenced plants had higher ROS accumulation than WT and *CaSLP*-knockdown pepper plants, thereby demonstrating the decreased *Pst.DC3000* resistance, as revealed by reduced cell death and bacterial numbers in pepper leaves compared with the control plants (Fig. 7C, G). To investigate the role of *CaSLP*-knockdown pepper plants, the bacterial numbers in *CaSLP*-silenced and control plants were measured. The bacterial numbers in *CaSLP*-silenced plants were higher than those in the control plants after *Pst.DC3000* treatment (Fig. 7D). Next, we measured the expressions of stress-related genes *CaNPR1, CaABR1,* and *CaPR1*. Further analysis showed that the relative expressions of *CaNPR1, CaABR1,* and *CaPR1* in *CaSLP* VIGS plants were strikingly lower than those of the control pepper plants (Fig. 7J-L). Collectively, these results illustrated that *CaSLP* plays a significant role in the plant’s response to *Pst.DC3000*.

### Enhanced resistance to *Pst.DC3000* in *CaSLP* transgenic Arabidopsis plants

**Fig. 8:**
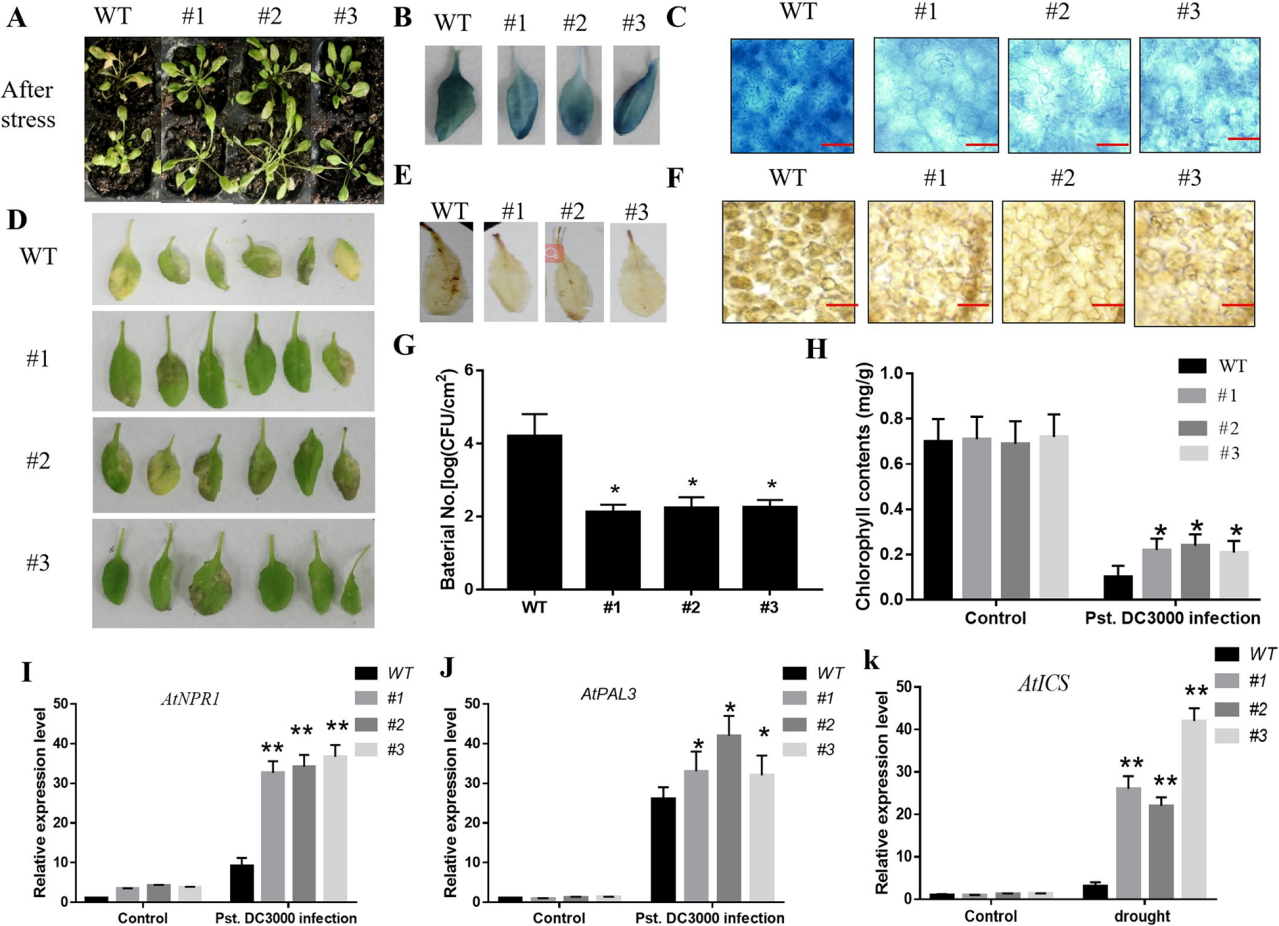
Overexpression of *CaSLP* confers enhanced resistance to *Pst.DC3000* stress in transgenic Arabidopsis. **A**, **D** Phenotype of transgenic and WT plants after the *Pst.DC3000* infection. Plants were infected with *Pst.DC3000* for 3 days. **B**, **E** Histochemical staining with 3,3’-diaminobenzidine (DAB) and Trypan blue for measuring the H_2_O_2_ contents of the transgenic and WT plants after 3 d of *Pst.DC3000* infection. Bar = 50 μm. **C**, **F** Trypan blue and DAB staining for cell death in the transgenic and wild-type (WT) plants after 3 d of *Pst.DC3000* infection. **G** Bacterial numbers. **H** Chlorophyll contents in the transgenic and WT plants after 3 d of *Pst.DC3000* infection. **I**-**K** The expressions of the SA response genes of *AtNPR1, AtPAL3,* and *AtICS*. Values are means ± SD (*n* = 3 replicates). Asterisks indicate a significant difference between the transgenic lines and WT under drought stress (*, *P* < 0.05; **, *P* < 0.01)

To explore the role of *CaSLP* in response to disease, the *CaSLP* transgenic lines and WT were infected with *Pst.DC3000.* 10 mM MgCl_2_ (control treatment) and *Pst.DC3000* suspensions were injected into the plant leaves. Under untreated conditions, the color of *CaSLP* transgenic lines and WT was almost the same. There was no substantial difference. Whereas, after 3 days post-*Pst.DC3000* injection, the WT showed more significant yellowing than the *CaSLP* transgenic lines. WT had lower chlorophyll content than the *CaSLP* transgenic lines (Fig. 8A, D, H). The bacterial counts of CaSLP transgenic lines and those treated with *Pst.DC3000* were then determined. In the *Pst.DC3000* treatment, the *CaSLP* transgenic lines showed lower bacterial numbers than WT, respectively (Fig. 8G). We also determined the DAB and Trypan blue staining. With *Pst.DC3000* treatment, the WT had higher values than the CaSLP transgenic lines (Fig. 8B, E). The transgenic lines had less ROS accumulation than WT, and *CaSLP* transgenic lines showed increased resistance to *Pst.DC3000*, as revealed by reduced cell death and bacterial numbers in Arabidopsis leaves compared with WT leaves (Fig. 8C, F). To further understand the underlying molecular mechanism of *CaSLP* in response to disease, we measured the expression levels of SA responses and the SA biosynthesis-related genes *AtNPR1, AtPAL3,* and *AtICS* after *Pst.DC3000* treatment. The expressions levels of *AtNPR1, AtPAL3,* and *AtICS* were greater in the transgenic plants than the WT plants (Fig. 8I-K). Our results indicated that *CaSLP* transgenic Arabidopsis improved the *Pst.DC3000* resistance.

### *CaSLP* enhances the binding of *CaNAC035* to its target gene promoters

**Fig. 9:**
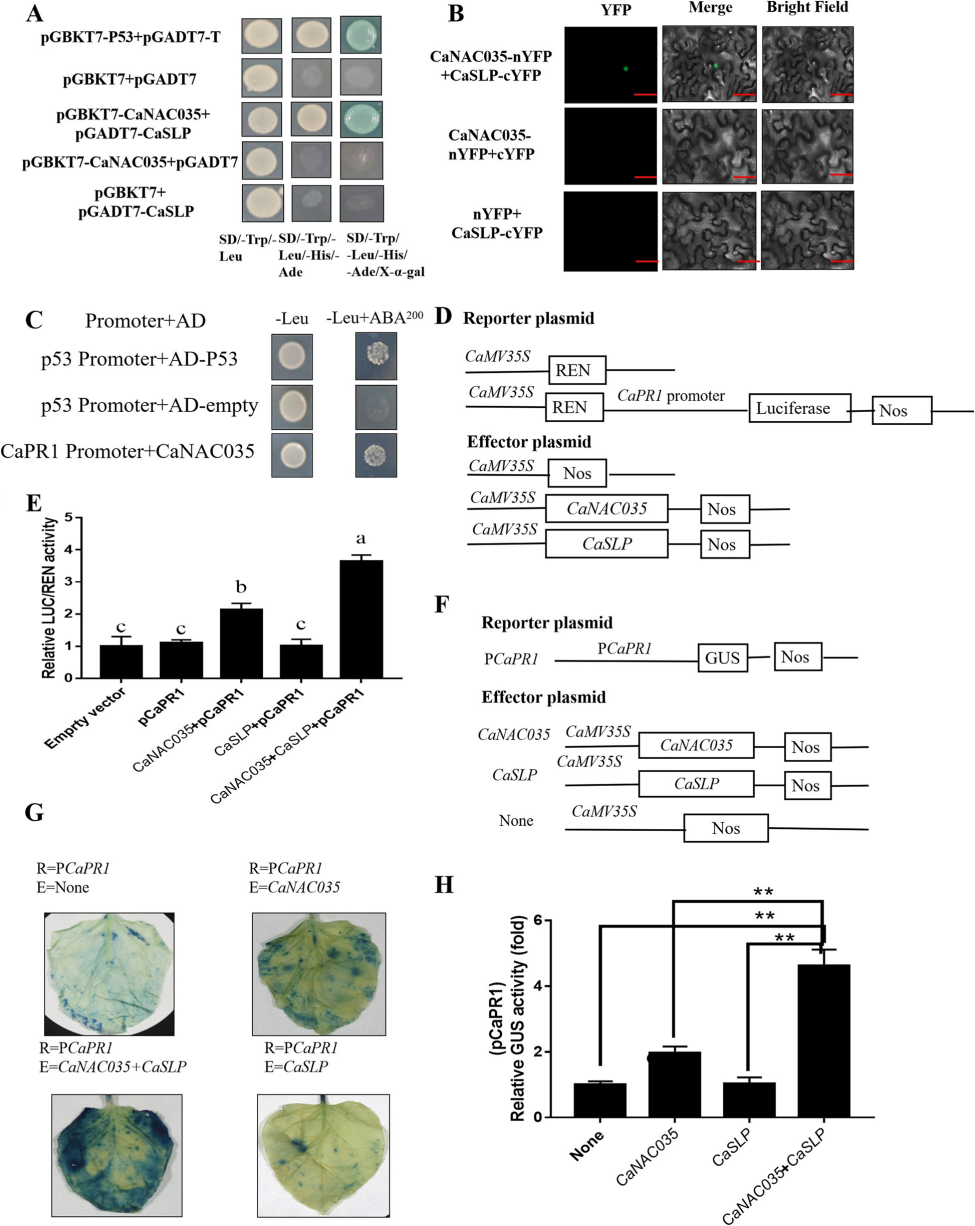
CaSLP enhances the binding of CaNAC035 to its target gene promoters. **A** Yeast two-hybrid assay of CaNAC035 and CaSLP. The full-length ones were CaNAC035 and CaSLP cloned into a pGBKT7 or a pGADT7 vector, respectively Yeasts grown in SD (-Trp/-Leu), SD (-Trp/-Leu/-His/-Ade) and SD (-Trp/-Leu/-His/-Ade + X-α-gal) media are indicated. **B** Bimolecular fluorescence complementation (BiFC) assay of CaNAC035 and CaSLP. A representative picture is shown here, GFP, green fluorescent protein. **C** Growth of yeast cells. **D**, **F** Diagrams of effector and reporter constructs. **E** luciferase (LUC)/Renilla luciferase (REN) activities detected from LUC/REN reporter system. **G**, **H** GUS activities assay the interaction of CaSLP and enhance the binding of CaNAC035 to the *CaPR1* promoter. Values are means ± SD (*n* = 3 replicates). Asterisks indicate significant difference (*, *P* < 0.05; **, *P* < 0.01)

The yeast two-hybrid (Y2H) method was used to analyze the target protein of CaNAC035. To examine the interaction of CaNAC035 with CaSLP, the Y2H and bimolecular fluorescence complementation (BiFC) assays were used in this study. The Y2H results showed that yeast containing CaNAC035 and CaSLP grew well on the selective solid medium of SD/-Trp/-Leu/-His/-Ade, and the yeast strains turned blue on the SD/-Trp/-Leu/-His/-Ade/X-a-gal solid medium (Fig. 9A). To further identify the interaction, we performed BiFC assays, which showed that coexpression of CaNAC0-nYFP with CaSLP-cYFP displayed significant fluorescence signaling in the cell nucleus (Fig. 9B). These results showed that CaNAC035 physically interacts with CaSLP in the cell nucleus. The transcripts of *CaPR1* were dramatically increased in the *CaNAC035*-To pepper (Fig. S2). We examined if CaNAC035 affects *CaPR1* transcription directly. NAC TFs can often bind to CACG. We analyzed the promoter of *CaPR1* and found that *CaPR1* had an NAC core-binding site. To determine the association between CaNAC035 and the *CaPR1* promoter, we carried out Y1H assays. The full length of *CaNAC035* was used as the prey, the *CaPR1* promoter fragment was used as bait. On medium without aureobasidin A (AbA) p53 promoter + AD-P53 (positive control), p53 promoter + AD-empty (negative control), and *CaPR1* promoter + AD-CaNAC035 grew normally. However, when 200 ng/ml AbA was added, the p53 promoter + AD-P53 (positive control) and CaPR1 promoter + AD-CaNAC035 survived, the p53 promoter + AD-empty (negative control) was inhibited. Yeast cells transformed transformed and showed full-length growth well on selective media. (Fig. 9C). These results showed a direct correlation of *CaNAC035* with the promoter of *CaPR1*. To further confirm the direct binding of *CaNAC035* to the *CaPR1* promoter, regulation of expression LUC/REN ratios was performed (Fig. 9D). Full-length of *CaNAC035* and *CaSLP* were inserted into pGreenII 62-SK to get an effector, full-length of *CaPR1* were inserted into pGreenII 0800-LUC to generate reporters. REN (Renilla luciferase) was used as an internal control for activity normalization. LUC:REN ratios were significantly elevated in *CaNAC035-*pGreen II 62-SK + *CaPR1-*pGreenII 0800-LUC and *CaNAC035-*pGreen II 62-SK + *CaSLP-*pGreen II 62-SK + *CaPR1-*pGreenII 0800-LUC than the control. Dual LUC assay results showed that the *CaPR1* could activate the CaNAC035 expression (Fig. 9E), indicating that *CaNAC035* is a direct upstream factor of *CaPR1*. GUS analysis was completed to explore the activity of *CaNAC035*pro with the addition of *CaPR1.* Transient expression assays with the β-glucuronidase reporter gene (GUS) in the leaves of tobacco (Nicotiana benthamiana) showed that *CaPR1* could activate the *CaNAC035* expression in vivo, and *CaSLP* can enhances the binding of *CaNAC035* to *CaPR1* promoter (Fig. 9F, G, H). These results *CaSLP* enhances the binding of *CaNAC035* to its target gene promoters.

### *CaSLP* regulates drought tolerance in a *CaNAC035*-dependent manner

**Fig. 10:**
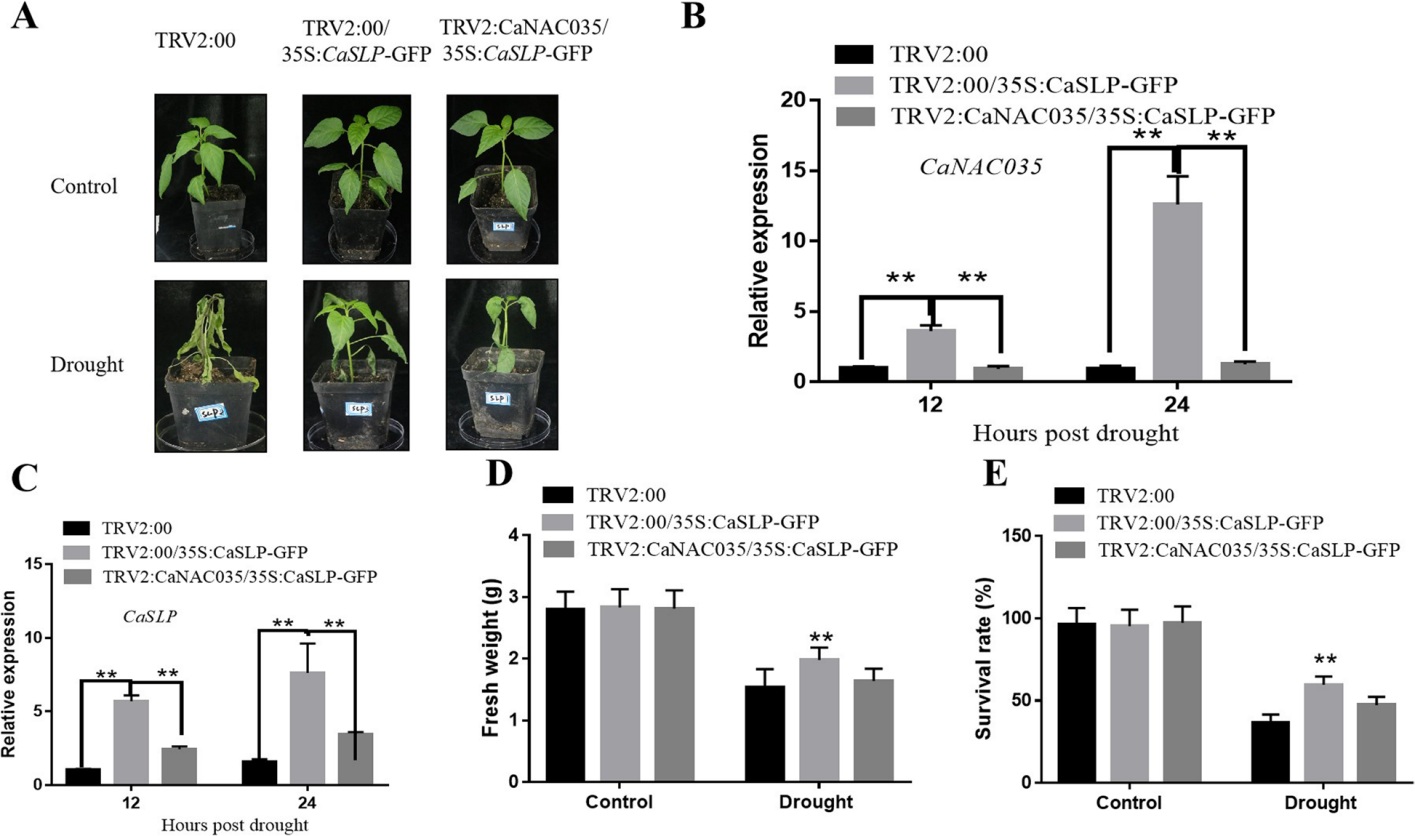
*CaNAC035* is required for *CaSLP*-mediated drought stress tolerance. **A** Phenotypes of *CaSLP*-silenced cells were coagroinoculated into 35S:*CaNAC035*:GFP. **B**, **C** The transcript levels of *CaNAC035* and *CaSLP* in TRV2:*CaNAC035*/35S:*CaSLP*:GFP, TRV2:00/35S:*CaSLP*:GFP, and TRV2:00/35S:GFP plants*.* **D** Fresh weight in TRV2:*CaNAC035*/35S:*CaSLP*:GFP, TRV2:00/35S:*CaSLP*:GFP, and TRV2:00/35S:GFP plants before and after 48 h of drought treatment. **E** Survival rates of TRV2:*CaNAC035*/35S:*CaSLP*:GFP, TRV2:00/35S:*CaSLP*:GFP, and TRV2:00/35S:GFP plants before and after 48 h of drought treatment. Values are means ± SD (*n* = 3). Asterisks indicate significant difference (*, *P* < 0.05; **, *P* < 0.01)

A previous study found that CaSLP interacts with CaNAC035 protein. To further verify the relationship between these two proteins and analyze whether *CaSLP* modulates drought stress in a *CaNAC035*-dependent manner, the *CaSLP*-silenced cells were inoculated into 35S:*CaNAC035*:GFP (Fig. 10A). qRT-qPCR showed that the expression levels of *CaNAC035* and *CaSLP* in TRV2:00/35S:*CaSLP*:GFP were more significant than those in TRV2:*CaNAC035*/35S:*CaSLP*:GFP and TRV2:00/35S:GFP at 12 and 24 h (Fig. 10B, C). Under drought stress, the TRV2:00/35S:*CaSLP*:GFP plants exhibited higher fresh weight and survival rate than the TRV2:*CaNAC035*/35S:*CaSLP*:GFP and TRV2:00/35S:GFP plants (Fig. 10D, E). Therefore, the CaNAC035 gene was necessary for *CaSLP*-mediated drought stress tolerance.

## Discussion

Water scarcity is a serious global barrier to agricultural production. Drought stress has a negative impact on plant growth, development, and distribution and results in yield reduction and economic loss (Kumar et al. 2019). Currently, it is an important evaluation index that is used to enhance crop drought tolerance (Dunn et al. 2019). *CaNAC035* plays a positive role in the plant’s tolerance to cold, salt and drought stresses (Zhang et al. 2020). In a previous study, using yeast two hybrid, we screened that 18 proteins that may interact with CaNAC035 (Zhang et al. 2020). In this study, we found that the *capsicum annuum* 40S ribosomal protein SA-like (CaSLP) is a CaNAC035-interacting protein in pepper (Fig. 9B). Subcellular location showed that CaSLP localized in the nucleus and cell membrane (Fig. 1A), and the results were consistent with its interaction location in vivo. MDA is a suitable index for lipid peroxidation when plants are subjected to stress (Mittler 2002). Electrolyte leakage is an important physiological index that identifies stress (Bajjiet al. 2002). In this study, the REL and MDA contents in *CaSLP* transgenic lines were significantly lower than those in WT under drought stress (Fig. 4). Our findings suggest that lipid peroxidation and membrane damage in drought-exposed *CaSLP* transgenic lines are lower than in drought those in the control group.

A high concentration of reactive oxygen species is damaging to plant cells and can destroy nucleic acid, oxidize protein, and cause lipid peroxidation. It is the primary factor impacting cell viability under abiotic stress (Gill and Tuteja 2010). Drought stress leads to the accumulation of ROS, which adversely affects plant growth and development (Miller et al. 2010). ROS is at a low level under normal conditions, but when plants are challenged by abiotic stress, ROS levels can rise sharply, leading to plant death. The accumulation of ROS is used to evaluate the capacity of plants subjected to stress. In this study, the data showed that *CaSLP-*To has lower H_2_O_2_ and O_2_^.−^ contents than the control after drought stress (Fig. 3). However, *CaSLP*-silenced pepper plants displayed the opposite effects, as shown by histochemical staining (DAB and NBT) (Fig. 2). *CaSLP*’s contribution to drought tolerance may be consistent with the ROS level and was through the regulation of antioxidant gene and maintenance of the homeostasis of reactive oxygen species. These findings indicated that transgenic plants had more antioxidant capacity than WT, which was due to considerably lower ROS levels and higher oxidative stress ability of the transgenic overexpression lines. VIGS plants had the reverse tendency. Another way that *CaSLP* improves drought tolerance is by more efficiently mobilizing the antioxidant system, which increases antioxidant capacity.

Stomata play a key role in regulating gas and water exchange during the stomatal development stage (McKown and Bergmann 2020). The stomata of plants are exposed to drought to prevent water loss (Schroeder et al. 2001). Increasing drought tolerance is connected with the stomatal closure of plants (Aubertet al. 2010). In this study, under drought stress, the *CaSLP*-silenced pepper plants had smaller stomatal apertures than the control plants (Fig. 2). To further know if the molecular mechanisms of *CaSLP* in response to drought stress were attributable to stomatal development genes, we tested the expressions of stomatal development genes. The stomatal development genes (*AtSDD1*, *AtYODA*, *AtFAMA*, *AtTMM,* and *AtMPK3*) of *CaSLP* transgenic Arabidopsis lines were significantly higher than those in WT plants (Fig. 6). These findings indicated that *CaSLP* contributes to drought tolerance and may be involved in stomatal regulation. However, the regulatory mechanism remains unclear. These results were consistent with those of previous reports. For instance, Arabidopsis *AtATAF1* enhances drought tolerance by reducing stomatal aperture (Wu et al. 2009). *AGL16* negatively modulates drought tolerance via stomatal movement in Arabidopsis (Zhao et al. 2020). *AtUNE12* confers salt tolerance by decreasing the stomatal aperture in Arabidopsis (He et al. 2022).

Exogenous spraying SA can increase plants’ ability to withstand drought and is crucial for plant abiotic stress tolerance (Antonić et al. 2016). SA can directly or indirectly induce some genes involved in abiotic stresses (Horváthet al. 2007). SA application slightly enhances the drought tolerance of *CaSLP*-silenced pepper (Fig. 5). Exogenous salicylic acid exhibits a good impact on these parameters, reducing the negative effects of water deficiency on plants while considerably reducing the water loss rate and chlorophyll content under drought stress (Purcarea et al. 2010). SA plays an important role in enhancing drought stress tolerance. To further determine whether the molecular mechanisms of *CaSLP* in response to drought stress were attributable to SA response maker genes. we determined the levels of SA response maker genes, including *AtNPR1, AtPAL3,* and *AtICS*. Among the SA response maker genes tested, the expressions of *AtNPR1, AtPAL3,* and *AtICS* dramatically increased in the *CaSLP*-OX compared with the WT (Fig. 8). Expression levels of SA response maker genes indicated that *CaSLP* may bind to the SA response maker genes promoter, resulting in enhanced *Pst.DC3000* resistance. Accumulating evidence indicated that *CaSLP* plays important roles in various stresses and might be related to SA signaling. These data revealed that *CaSLP* plays a role in the response to *Pst.DC3000* stress resistance by participating in the SA signaling pathway.

Based on the Y1H, LUC/REN, and GUS results, CaSLP interacts with CaNAC035 and synergistically enhances the transcriptional activity of *CaPR1 *(Fig. 9), which indicates its key role in the regulation of stress resistance. The regulatory pathway explains *CaSLP* response to stress tolerance. SA is an important signaling factor in plant stress and is involved in some important physiological and biochemical processes in plants. It also plays a diverse role in plant stress response in the form of signal molecules. SA plays a diverse role in the regulation of resistance to abiotic stress by enhancing the binding ability of SA and its receptor protein, which can perceive and transmit stress signals.

**Fig. 11:**
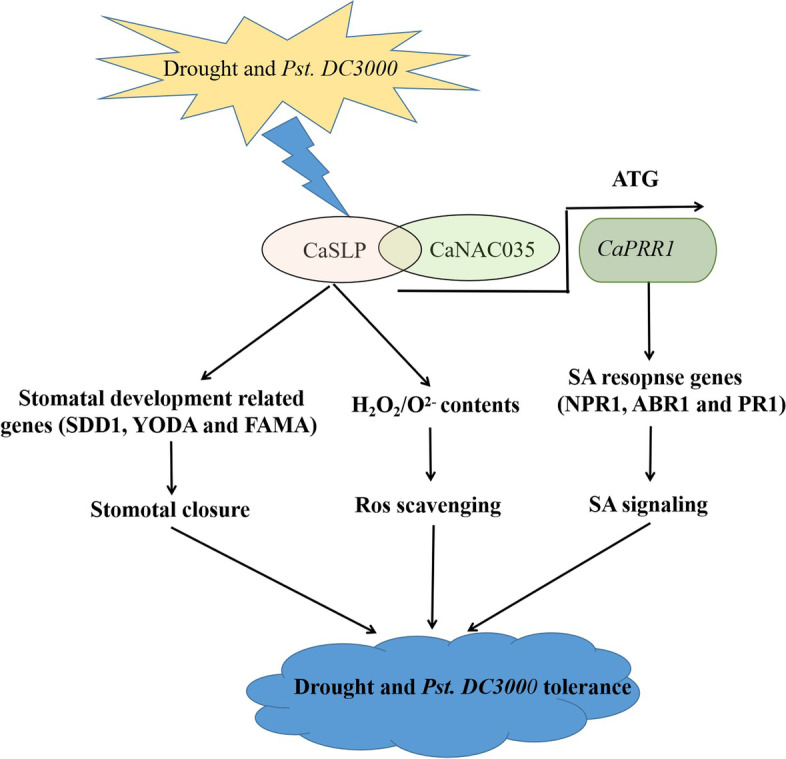
In this model, Proposed model for *CaSLP* in response to drought and *Pst.DC3000* tolerance stress through three main mechanisms: (1) By interacting with CaNAC035, *CaSLP* alleviates water loss by stimulating stomatal closure and reducing stomatal density; (2) Upon exposure to drought and SA stresses, *CaSLP* mediated drought and *Pst.DC3000* resistance stress was cleared by ROS; (3) CaNAC035 interacts with CaSLP, and *CaNAC035* acts as a transcriptional activator binds to the promoter of *CaPR1* to modulate the drought and *Pst.DC3000* resistance

In conclusion, the transcription factor CaNAC035 interacted with CaSLP in the nucleus, and *CaSLP* played a positive role in drought and *Pst.DC3000* stress resistance in pepper. We proposed the model for *CaSLP* in response to drought and *Pst.DC3000* resistance stress (Fig. 11).

## Methods

### Plant material and growth conditions

Pepper (*Capsicum annuum* L., ‘P70’) and Arabidopsis (Columbia) seeds were obtained from the Laboratory of Vegetable Plant Biotechnology and Germplasm Innovation, Northwest A&F University, Yangling, China. The pepper plants were cultivated with a light cycle of 16 h light/8 h dark and 22 °C light /18 °C dark cycle with 75% relative humidity. The Arabidopsis seeds were sterilized before being cultivated on Murashige Skoog (MS) solid medium and vernalized for 1 day at 4 °C. The plants were restored to regular conditions after 7 days (22 °C with a photoperiod of 16 h light/8 h dark).

### RNA extraction and qRT-PCR analysis

The total RNA was extracted according to the manufacturer’s instructions using a Tiangen RNA extraction kit (Beijing, China) and the Synthesis of cDNA was performed using a cDNA Synthesis Kit (Takara). The qPCR was conducted on the Applied Biosystems instrument using the SYBR Green Master Mix, following the manufacturer’s protocol (Ding Ning). The quantitative real-time polymerase chain reaction (qRT-PCR) primers were designed by using NCBI (https://www.ncbi.nlm.nih.gov/) and the primer was shown in Table S1. The relative expression was calculated using the 2^−ΔΔCT^ method (Zhuo et al. 2013). Three biological replicates were used for this study.

### *CaSLP* gene function identification

To generate a transient expression of *CaSLP* in pepper leaves, the coding region (CDS) of *CaSLP* was cloned and introduced into the pVBG2307::GFP vector. Then the 35S:*CaSLP*:GFP and 35S::GFP were transformed into *A. tumefaciens* strain GV3101. Transient expression in pepper leaves was performed as described by Huang et al. 2020. Leveraging the virus-induced gene silencing technique, *CaSLP*-silenced plants were grown. The pTRV1 and pTRV2 vectors were used for this study, and a 300-bp fragment of *CaSLP* was cloned and inserted into the pTRV2 vector. The pTRV1, pTRV2 (control), and pTRV2:*CaSLP* were separately transformed into *A. tumefaciens* strain GV3101. The infection method was followed as described by previous study Dai et al. 2018. The infected pepper plants were cultivated in a light/dark cycle of 22 °C/18 °C. After 28 days, we collected DNA and RNA to analyze the *CaSLP* transcript levels and identified positive plants using a specific primer.

### *CaSLP*-OX expression in transgenic Arabidopsis plants

For Arabidopsis transformation, the full length of *CaSLP* was cloned and combined into the pVBG2307:GFP vector. Then, the 35S:*CaSLP*:GFP fusion vector was transformed into *A. tumefaciens* strain GV3101. For transformation, the floral dip method was used as in previous reports (Clough and Bent 1998). Then, the transgene plants were screened on 1/2 MS solid medium, which contained 50 mg/ml kanamycin. We extracted DNA and RNA from T3 transgenic Arabidopsis lines to analyze the transcript levels of *CaSLP* and also confirmed the positive plants. For this experiment and future research, T3 homozygous lines were chosen.

### Protein interaction assays

The yeast two-hybrid assay was used for the experiment. In the pGBKT7 vector as bait plasmid, the complete length of *CaNAC035* was introduced, and the full length of *CaSLP* was placed into the pGADT7 vector as prey plasmid. The recombinant vector was transformed into yeast Y2H. The strains were cultivated on SD (-Trp/-Leu), SD (-Trp/-Leu/-His/-Ade) and SD (-Trp/-Leu/-His/-Ade + X-α-gal) media for 3 days. Bimolecular fluorescence complementation (BiFC) was conducted as previously reported (Choi et al. 2012). To identify the proteins that can interact with a DNA sequence of interest, Y1H library screening was performed by using the Matchmaker Gold Yeast One-Hybrid Library and Screening kit (Clontech,CA, USA). The full-length CDS of *CaNAC035* was placed into the pGADT7 vector to function as a prey vector. The promoter portions of *CaPR1* were added to the pAbAi vectors. Recombinant plasmids were cotransformed into the Y1H yeast strain following the instructions provided by the manufacturer (Clontech, USA). For 3 days, the yeast strains were cultured on SD/Leu and SD/Leu/AbA media. The assay was similar to the widely used yeast two-hybrid assay that identifies protein protein interactions in small- or large-scale settings.

### Drought stress and pathogen inoculation and disease symptom assays

To assess drought tolerance in pepper plants, the leaves of *CaSLP*-silenced and control pepper plants were subjected to a 15-day drought. Leaves of pepper were collected at different stages after drought treatment. To further identify the role of *CaSLP* in transgenic Arabidopsis drought stress tolerance, 3-week-old T3 transgenic and WT lines were used in the experiments. The T3 transgenic and WT lines were treated with drought treatment for 10 d. Samples for gene expression analysis were collected, and phenotypic changes were observed and photographed. Following the drought treatments, samples were collected to investigate stress-related gene expression and physiological indicators. Well-watered plants were used as the positive control. The bacterium *Pst.DC3000* was incubated overnight in Kings B solid medium containing 25 mg mL^−1^ rifampicin at 28℃ and then resuspended in 10 mM MgCl_2_. The whole plant was inoculated with a bacterial suspension of 10^7^ colony- forming units (CFU) mL^−1^ and 0.02% Silwet L-77 (Li et al. 2015). Trypan blue staining and bacterial population counts (CFU) were performed 3 days after inoculation to evaluate disease symptoms (Bai et al. 2012; Wolfe et al. 2000).

### Assessment of physiological characteristics and application of a salicylic acid spray

The malondialdehyde (MDA) content was performed essentially as previously described (Liu et al. 2006), the relative electrolyte leakage (REL) was determined based on an earlier study (Dahro et al. 2016), the water loss rate was examined according to the protocol of Zhang et al. (2022), the accumulation of H_2_O_2_ and O_2_^.−^ contents were conducted by histochemical staining of 3,3’-diaminobenzidine (DAB) and nitro-blue tetrazolium (NBT), respectively, using the method of Wang et al. (2011) O_2_^.−^ the content was examined based on the method of Ma et al. (2016), the H_2_O_2_ measurement was determined as described by Geng and Liu (2018). The stomatal aperture assay was performed as described by Jiang et al. (2014). The stomatal index was determined as the number of stomata divided by the total number of epidermal cells. For the application of an salicylic acid spray, *CaSLP*-silenced and control plants were exogenously given 2 mM salicylic acid, and the plants were exposed to drought to determine the effects of exogenous salicylic acid on *CaSLP*’s response to drought. Exogenous treatment with 2 mM salicylic acid was performed for 2 days. We measured the growth performance and calculated the physiological index. We added Tween-20 (0.05%) for exogenous spraying of salicylic acid on *CaSLP*-silenced and control plants.

### Dual luciferase and GUS activities assays

To rapidly and accurately determine the activity of a given promoter, the *CaPR1* promoter elements were introduced into a pGreen62-SK vector. After being placed into a pGreen0800-luciferase (LUC) vector, *CaNAC035*’s coding sequences were obtained. Four-week-old tobacco leaves were injected with the recombinant vectors after being cotransformed into *Agrobacterium* Gv3101 bacteria (Nicotiana benthamiana). Using the Dual-Luciferase® machine, the ratio of luciferase (LUC) and Renilla (REN) was computed to calculate transient expression (Promega, WI, USA). Transient GUS activity was determined with tobacco leaves. The *CaPR1* promoter was inserted into pCAMBIA1300-GUS to activate the GUS reporter gene. The ORFs of *CaSLP* and *CaNAC035* were cloned into the pVBG2307:GFP plasmid to obtain 35S:*CaSLP* and 35S:*CaNAC035* recombinant plasmids, respectively. Different combinations were injected into tobacco leaves (5 weeks old) for *Agrobacterium*-mediated transformation. The injected tobacco seedlings grew for approximately 2 days under normal conditions. The GUS operations were also carried out as indicated by (Ma et al. 2021).

### Statistical analysis

One-way analysis of variance (ANOVA) tests with significant differences of P 0.05 (*) and P 0.01 (**) were used for statistical studies in the statistical software SPSS (version 21.0, USA).

### Supplementary Information

**Additional file 1: Supplementary Figure S1.** Phenotypes and silencing efficiency of *CaSLP* in silenced and control plants. **Supplementary Figure S2.** Expression levels of SA response genes in *CaNAC035*-To and control plants. **Supplementary Table S1.** Primers were used for the qRT-PCR.

## Data Availability

The data that support the results included in this article and its supplementary materials. Other relevant materials are available from the corresponding author upon reasonable request.

## Abbreviations

SA: Salicylic acid
Y2H: Yeast two-hybrid
BiFC: Bimolecular fluorescence complementation
