# Supplementary material for: Salicylic acid-related ribosomal protein CaSLP improves drought and Pst.DC3000 tolerance in pepper
Source: Mol Hortic. 2023 Mar 14;3:6. doi: 10.1186/s43897-023-00054-3 (PMC10514951; doi:10.1186/s43897-023-00054-3)
Supplement: Supplementary file 1 — Additional file 1: Supplementary Figure S1. Phenotypes and silencing efficiency of CaSLP in silenced and control plants. Supplementary Figure S2. Expression levels of SA response genes in CaNAC035-To and control plants. Supplementary Table S1. Primers were used for the qRT-PCR. [file 43897_2023_54_MOESM1_ESM.docx]

supplementary materials:


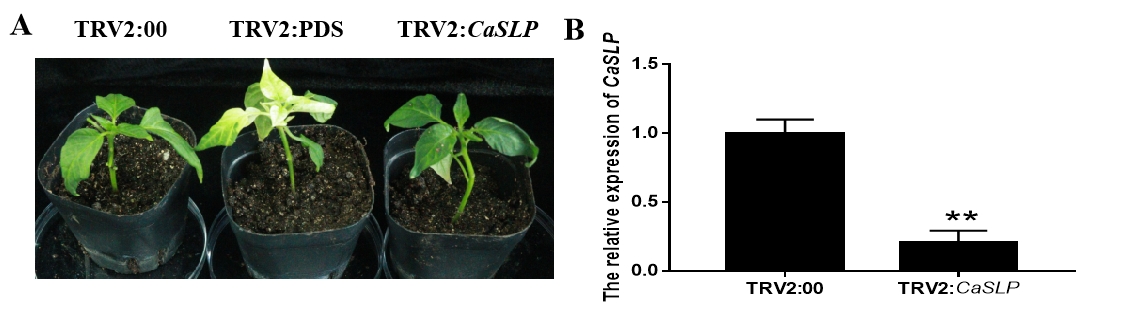
Figure S1

**Figure S1** Phenotypes and silencing efficiency of *CaSLP* in silenced and control plants. A, The phenotypes of *CaSLP*-silenced plants. B, qRT-PCR was used to analyze the *CaSLP* expression of TRV2-*CaSLP* plants, using. Actin was used as an internal control.

Figure S2

**Figure S2**. Expression levels of SA response genes in CaNAC035-To and control plants.

**Table S1**. Primers were used for the qRT-PCR.

| Order number | Primer Abbreviation | Primer Sequence (5’- 3’) |
| --- | --- | --- |
| 1 | AtMPK3-F | CGGCAACTTCCCAACTTCCCA |
| 2 | AtMPK3-R | GAGTGCTATGGCTTCTTGGTAG |
| 3 | At PR1-F | ACGGGGAAAACTTAGCCTGG |
| 4 | At PR1-R | TTGGCACATCCGAGTCTCAC |
| 5 | AtAtICS1-F | CTTCCGTGACCTTGATCCTTTCT |
| 6 | AtAtICS1-R | CAGCGATCTTGCCATTAGGATC |
| 7 | AtPAL3-F | AGGCTACTCT GGTATACGCT TTGAGATCCT |
| 8 | AtPAL3-R | GATACTCCAGCGAGCTTGAAGGCCT |
| 9 | AtSDD1-F | CAACGGTCGTATTTTCCTATTCA |
| 10 | AtSDD1-R | AGACATGAACTATATTAACTGAAG |
| 11 | AtYODA-F | TCTGTCTGCATCTCCTAGGCGG |
| 12 | AtYODA-R | CTGAGTAGCCATATCTCCACCA |
| 13 | AtFAMA-F | GGATTGACCCCGTTTATTTCTTG |
| 4 | AtFAMA-R | CTTCCTCTTGCTCTTCACCTCC |
| 15 | AtTMM-F | ACAAACTGGATTCACGATGACC |
| 16 | AtTMM-R | CGTAAGAGCGTTGTGGATCAC |
| 17 | AtActin-F | GTCGTACAACCGGTATTGTGCT |
| 18 | AtActin-R | TGTCTCTTACAATTTCCCGCTCT |
| 19 | AtActin2-F | GGTAACATTGTGCTCAGTGGTGG |
| 20 | AtActin2-R | AACGACCTTAATCTTCATGCTGC |
| 21 | CaNPR1-F | ACTTCTTCGCCGACGCCAAG |
| 22 | CaNPR1-R | GCCAACACATTCACCAGAGCATC |
| 23 | CaPR1-F | GCCGTGAAGATGTGGGTCAATGA |
| 24 | CaPR1-R | TGAGTTACGCCAGACTACCTGAGTA |
| 25 | CaABR1-F | ACATGTCGGAGAACTCGGTG |
| 26 | CaABR1-R | TATCTTGTGCCTGTGTGCGT |
| 27 | CaUBI3-F | TGTCCATCTGCTCTCTGTTG |
| 28 | CaUBI3-R | CACCCCAAGCACAATAAGAC |
| 29 | CaActin2-F | TCCACCTCTTCACTCTCTGCTC |
| 30 | CaActin2-R | TGACCCATCCCTACCATAACAC |
